# ORTHOSCOPE Analysis Reveals the Presence of the Cellulose Synthase Gene in All Tunicate Genomes but Not in Other Animal Genomes

**DOI:** 10.3390/genes10040294

**Published:** 2019-04-10

**Authors:** Jun Inoue, Keisuke Nakashima, Noriyuki Satoh

**Affiliations:** Marine Genomics Unit, Okinawa Institute of Science and Technology Graduate University, Onna, Okinawa 904-0495, Japan; keisuke@oist.jp (K.N.); norisky@oist.jp (N.S.)

**Keywords:** tunicates, *CesA* genes, ORTHOSCOPE, horizontal gene transfer

## Abstract

Tunicates or urochordates—comprising ascidians, larvaceans, and salps—are the only metazoans that can synthesize cellulose, a biological function usually associated with bacteria and plants but not animals. Tunicate cellulose or tunicine is a major component of the outer acellular coverage (tunic) of the entire body of these organisms. Previous studies have suggested that the prokaryotic cellulose synthase gene (*CesA*) was horizontally transferred into the genome of a tunicate ancestor. However, no convenient tools have been devised to determine whether only tunicates harbor *CesA*. ORTHOSCOPE is a recently developed tool used to identify orthologous genes and to examine the phylogenic relationship of molecules within major metazoan taxa. The present analysis with this tool revealed the presence of *CesA* orthologs in all sequenced tunicate genomes but an absence in other metazoan genomes. This supports an evolutionary origin of animal cellulose and provides insights into the evolution of this animal taxon.

## 1. Introduction

In the 4th century BC, Aristotle described an invertebrate animal group called ascidians. Their characteristic feature is that the entire adult body has a thick, unique covering (translated in a previous study [1]). This outer layer, which is called the tunic or test, is non-cellular, like the shell of bivalves. Because of the presence of the covering and the morphological feature of the pharyngeal gills, ascidians and salps, and later larvaceans, were given the name Tunicier or Tunicata [2,3]. Tunicates have also been called urochordates on the basis of their chordate origins and their evolution together with cephalochordates and vertebrates [4,5]. Nevertheless, the tunic has been recognized as a feature specific to this animal group. Cellular and biochemical studies isolated a molecular component of the tunic, which was named tunicine [6]. Surprisingly, tunicine turned out to be a form of cellulose [7], a carbohydrate class formerly associated with bacteria and plants but not animals. Since then, the evolutionary origin of animal cellulose has piqued the interest of researchers with regard to the evolution of tunicates.

Cellulose is a polysaccharide consisting of linear chains of β1,4-linked d-glucose units. The dominant enzyme involved in cellulose production is cellulose synthase (CesA), membrane-embedded glycosyl transferase (GT). In 2004, two laboratories independently reported *CesA* in the ascidians *Ciona savignyi* (*Cs-CesA*) [8] and *Ciona intestinalis* (*Ci-CesA*) [9], and later two copies were found in a larvacean, *Oikopleura dioica* (*Od-CesA1* and *2*) [10,11]. The role of *Ci-CesA* in cellulose biosynthesis is evident from a mutant in which the enhancer element of the gene is a transposon-mediated mutation that results in the absence of cellulose biosynthesis activity [12].

**Figure 1:**
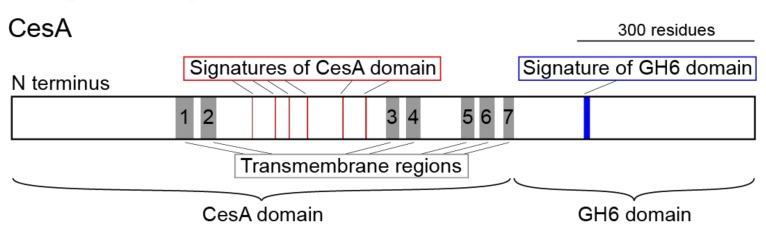
The composition of tunicate CesA, shown by a schematic of the domains. Tunicate CesA is a seven transmembrane (TM) domain protein, and a fusion protein of CesA and GH6 domains that are separated at the end of TM7. Red indicates sequences used for CesA domain identification and blue indicates sequences used for GH6 domain identification. Molecular signatures of CesA and GH6 domains follow Nakashima et al. [10].

The tunicate *CesA* is a large gene; *Ci-CesA*, for example, is composed of 21 exons, covers ~14 kb of the genome, and encodes a protein of 1277 amino acids with seven transmembrane domains (Figure 1). Interestingly, tunicate *CesA* produces a fusion protein that contains both a CesA domain belonging to glycosyl transferase family 2 (GT2) and a cellulase domain belonging to glycosyl hydrolase family 6 (GH6) (Figure 1). Such atypical fusion genes are not found in public databases, suggesting that the two domains originated from two distinct genes. In fact, in some *Streptomyces* genomes, the *CesA* and *GH6* genes are present as individual genes but are located closely [9]. Molecular phylogenetic analyses, however, demonstrated that the CesA and GH6 domains included neither eukaryote nor bacterial clades ([10,11]), leading researchers to wonder how the tunicate ancestor acquired this unique *CesA* [13].

Among the many questions about tunicate *CesA* genes, the most basic is whether *CesA* is present only in tunicate genomes or is also present in genomes of other metazoans. This question had not yet been addressed owing to a lack of appropriate tools. We recently developed a web tool called ORTHOSCOPE [14] (http://orthoscope.jp or https://github.com/jun-inoue/orthoscope) that allows users to identify orthogroups of specific protein-coding genes within ~36 bilaterian lineages. Because ORTHOSCOPE is ideal for determining the presence or absence of *CesA* orthologs in morphologically and genetically diverse metazoans, we here sought to answer this evolutionary question.

## 2. Methods

### 2.1. Data Collection

ORTHOSCOPE v.1.0.2 [14] was used to collect *CesA* ortholog candidates in tunicate and non-tunicate organisms. Gene models were selected from 49 species, including a bacterium, a land plant, a fungus, an ichthyosporean, two choanoflagellates, and 43 metazoans from sponges to vertebrates (Table 1). The metazoan species included 21 animal taxa (Table 1). All data sources are available at http://orthoscope.jp. The eight tunicate species examined were *Botrylloides leachii* [15], *Botryllus schlosseri* [16], *Ciona intestinalis* [17], *C. savignyi* [18], *Molgula occidentalis* [19], *M. oculate* [19], *Oikopleura dioica* [20], and *Salpa thompsoni* [21]. Amino acid sequences of tunicate CesA are shown in Supplementary Figure S1.

The CesA domain (2487–3072 bp before TM7 [pfam13641]; Figure S1) and GH6 domain (1233–1371 bp after TM7 [pfam01341]) included in full-length (3756–4443 bp) coding sequences of *C. intestinalis* (Ci-CesA), *O. dioica* (Od-CesA1 and 2), and *M. tectiformis* (Mt-CesA) were used as queries. The following parameters were used: Mode = Tree Search Only, Focal group = Deuterostomia, E-value threshold = 1e−3, Number of hits to report per genome = 3, Dataset = DNA (Exclude 3rd). Collected sequences were downloaded and used in the subsequent phylogenetic analyses.

### 2.2. Molecular Phylogenetic Analysis

Although ORTHOSCOPE automatically generates neighbor-joining trees, for more precise analyses, maximum-likelihood (ML) trees were estimated based on downloaded sequences using a pipeline distributed from the ORTHOSCOPE instruction site. The pipeline automatically aligns amino acid and coding sequences and estimates gene trees, as below. Protein sequences were aligned using MAFFT v7.407 [22]. The aligned protein sequences were trimmed by removing poorly aligned regions using TRIMAL v1.2 [23] with the option “gappyout”. Corresponding coding sequences were aligned using PAL2NAL v14 [24] with reference to the protein alignment and used for estimating gene trees. Unambiguously aligned sequences were divided into two partitions (first and second codon positions), and the dataset was subjected to the partitioned ML analysis using RAxML 8.2.6 [25]. The best-scoring ML tree was estimated using a general time reversible [26] + γ model [27] of sequence evolution (the model recommended by the author) with 100 bootstrap replicates. For tree rooting, ORTHOSCOPE selects the lowest BLAST hit of most distantly related species from query species in the ORTHOSCOPE taxonomic list. For phylogenetic analyses, the following sequences were used: *S. coelicolor* (Ensembl Bacteria 42: CAB44539, CAB65566, CAB65568, and CAB72208), *A. thaliana* (Ensembl Plants 42: AT2G24630.1, AT3G07330.2, AT3G28180.1, AT4G07960.2, and AT4G31590.1), *M. brevicollis* (NCBI: XP001745167.1), *M. leidyi* (Ensembl Metazoa 38: ML10106a-RA), *O. bimaculoides* (Ensembl Metazoa 38: Ocbimv22016613m), *O. dioica* (NCBI: AB543593.1 and AB543594.1; OikoBase [http://oikoarrays.biology.uiowa.edu/Oiko]: GSOIDG00009745001, GSOIDG00010490001, and GSOIDG00017000001), *O. longicauda* (NCBI: AB543515.1 and AB543515.1), *S. thompsoni* (NCBI: GFCC01072613.1.p1, GFCC01117283.1.p1, and GFCC01119318.1.p1), *H. roretzi* (NCBI: AB543517.1), *B. leachii* (aNISEED [https://www.aniseed.cnrs.fr/aniseed]: v3.S133.g02304.01.p and v3.S157.g03251.01.p), *B. schlosseri* (*B. schlosseri* genome project [http://botryllus.stanford.edu/botryllusgenome/download]: 008441g44193, 008441g44194, 008489g44331, 008489g44332, 008778g45080, and 010729g9326), *M. occidentalis* (aNISEED: S285391.g07021.01.p, S469068.g15913.01.p, S469068.g15914.01.p, and S469068.g15915.01.p), *M. oculata* (aNISEED: v1-2.S112948.g12660.01.p, v1-2.S69739.g04625.01.p, and v1-2.S71617.g04842.01.p), *M. tectiformis* (NCBI: AB543516.1), *C. savignyi* (Ensembl 91: ENSCSAVT00000016381.1), and *C. intestinalis* (NCBI: BAD10864; Ensembl 91: ENSCINT00000032307.1 and ENSCINT00000007306.3).

## 3. Results and Discussion

### 3.1. CesA is Present in the Genomes of All Tunicates but Not in the Genomes of the Other Metazoans Examined

**Figure 2:**
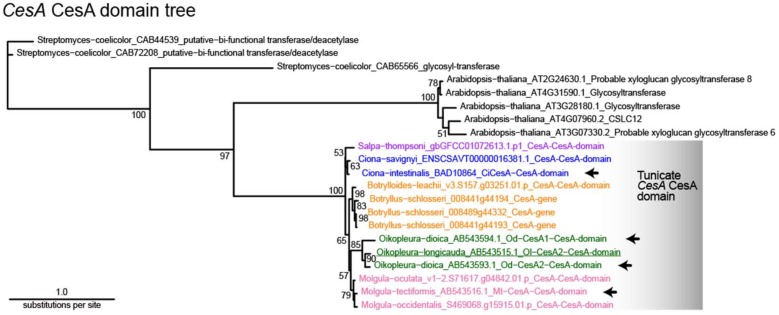
Molecular phylogenic tree of the CesA domain obtained using ORTHOSCOPE analysis (884 nucleotide sites). The CesA domains of four *CesA* genes from three tunicates (indicated with arrows) were used as query sequences. The *Streptomyces* sequence (CAB44539) was selected for tree rooting. Because all the non-tunicate metazoans examined did not contain *CesA* orthologs, the tree includes those of *Streptomyces* and *Arabidopsis* in addition to tunicates. The numbers beside the nodes indicate bootstrap probabilities (>50%). To count orthologs, identical sequences derived from ORTHOSCOPE were replaced with query sequences. Sequences ending with “-gene” are raw data, whereas those ending with “-domain” are partial gene sequences. Color coding for taxonomic groupings is used in the other figures. Underlined sequences were manually added to the sequence alignment.

**Figure 3:**
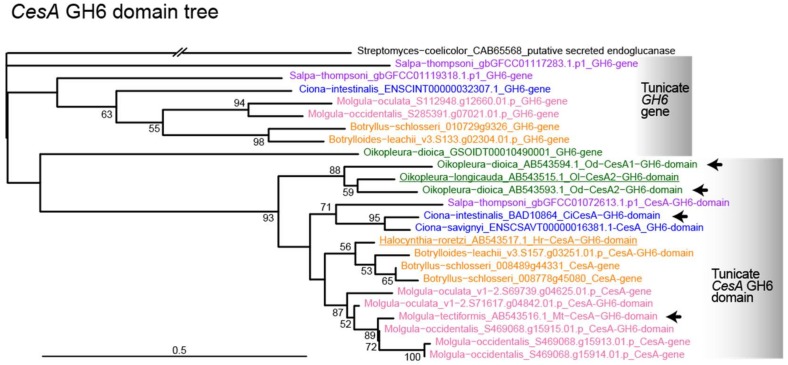
Molecular phylogenic tree of the GH6 domain obtained using ORTHOSCOPE analysis (438 nucleotide sites). The GH6 domains of four *CesA* genes of three tunicates (indicated with arrows) were used as query sequences. The *Streptomyces* gene (CAB65568) was selected for tree rooting. Because all the non-tunicate metazoans examined do not contain *GH6* orthologs, the tree includes only tunicates. It should be noted that tunicate genomes contain not only *CesA* genes, including the GH6 domain, but also another tunicate-specific *GH6* gene.

The presence or absence of tunicate *CesA* ortholog has not been extensively examined in genomes of non-tunicate metazoans, although horizontal gene transfers of cellulases were known in plant-parasitic nematodes [28,29]. As shown in Table 1, we used ORTHOSCOPE v.1.0.2 to identify candidates of *CesA* orthologs in a bacterium, a land plant, a fungus, an ichthyosporean, two choanoflagellates, a porifera, a placozoan, three cnidarians, a ctenophore, a platyhelminthes, an annelid, a nemertean, a brachiopod, a cephalopod, a gastropod, three bivalves, two nematodes, a merostomata, a chilopod, a malacostracan, seven insects, two echinoderms, two hemichordates, two cephalochordates, two vertebrates, and eight tunicates. The coding sequences for the CesA (GT2) domains and GH6 domains of the four tunicate *CesA* genes were each used as queries (Figure 2 and Figure 3).

The analysis showed that the tunicate *CesA* GH6 domain sequence is present in all eight species of tunicates (Table 1; GH6 column), but not in the other 35 metazoan species. Because these species cover diploblasts, protostomes, and deuterostomes, we concluded that the *CesA* GH6 domain is specific and unique to tunicates among metazoans. As mentioned above, the presence of the *GH6* gene is reported in fungi and bacteria, including *Streptomyces* [10,11], but not reported in available metazoan genomes. Supporting this, we did not find *CesA* GH6 domain orthologs not only in non-urochordate metazoans but also in the five non-metazoan eukaryotes examined here (Table 1).

The results of the ORTHOSCOPE search with the CesA domain of tunicate *CesA* were not as straightforward. First, we found the presence of the CesA domain in all eight species of tunicates (Table 1). It has been shown that the land plant *Arabidopsis thaliana* produces cellulose for components of its cell wall, but the fungus *Saccharomyces cerevisiae* does not produce cellulose. We confirmed the presence of the CesA domain in *A. thaliana* and its absence in *S. cerevisiae* (Table 1).

We did not find the CesA domain in most of the metazoan and three non-metazoan eukaryote lineages (Table 1). Exceptions were the choanoflagellate *Monosiga brevicollis*, the ctenophore *Mnemiopsis leidyi*, and the cephalopod *Octopus bimaculoides* (Table 1). In these cases, ORTHOSCOPE detected sequences that resemble CesA domains of the tunicates. Therefore, we examined the BLAST hits from *Monosiga* (M-b-XP001745167.1), *Mnemiopsis* (M-l-ML 10106a-RA), and *Octopus* (Ocbimv22016613m). Including these BLAST hits of *Monosiga*, *Mnemiopsis*, and *Octopus*, we constructed a molecular phylogenetic tree of *CesA* (Figure S2) with BLAST hits consisting of full-length *CesA*. The resulting tree showed that the branch lengths of the CesA domain of *Monosiga*, *Mnemiopsis*, and *Octopus* are comparatively long. Their lengths are in contrast to those of the tunicate CesA domains, which cluster with short branch lengths. Therefore, we concluded that the CesA-like domains of *Monosiga*, *Mnemiopsis*, and *Octopus* are not true orthologs of the tunicate CesA domain.

To examine this issue further, we carried out an ORTHOSCOPE analysis using the *Monosiga* M-b-XP001745167.1 as a query. This analysis pulled out resembling sequences from many metazoans, including echinoderms, hemichordates, and vertebrates (Figure S3). However, these hits consisted of THOC7 or uncharacterized proteins and did not generally resemble the CesA domain. One exception was the similarity of M-b-XP001745167 to *Oikopleura* CesA-1 protein. Reciprocal BLAST searches sometimes extract those with sequence similarities but not truly orthologous genes [30], and this similarity may represent such a case.

Based on results of both CesA domain and GH6 domain analyses, we concluded that *CesA* is present exclusively in tunicates but is absent in all other metazoans. This conclusion supports a previous evolutionary note that tunicate *CesA* was derived via horizontal transfer from bacterial *CesA* genes [8,9]. To infer the origin of tunicate CesA or GH6 domains of *CesA* gene outside metazoans, denser taxonomic samplings from non-metazoan eukaryotes are needed.

### 3.2. Molecular Phylogenetic Analysis with the CesA Domain

The ORTHOSCOPE run created a molecular phylogenetic tree of the CesA domain (Figure 2) by conducting BLAST search against gene models of six non-metazoans and 43 metazoans, including eight tunicates. The ML tree was constructed with a *Streptomyces* gene (CAB44539) as an outgroup. Because the CesA domain was identified only in *Streptomyces*, *Arabidopsis*, and tunicates, the resultant tree is composed of only the three groups.

In the tunicate lineage, the *Oikopleura* CesA domains showed longer branches than those of the other tunicates. Previous studies showed that the rate of molecular changes is faster in *Oikopleura* than in other tunicates, causing a long-branch attraction in the tree creation (e.g., [31]). Thus, the phylogenetic position of larvaceans among tunicates is still unresolved [32]. In Figure 2, the *Oikopleura* CesA domain was clustered with the *Molgula* CesA domain, to which *Botrylloides*/*Botryllus* CesA formed a sister group. In contrast, the CesA domains of *Ciona* and *Salpa* formed another distinct clade within the entire tunicate group. 

### 3.3. Molecular Phylogenetic Analysis with the GH6 Domain

Figure 3 shows the phylogenetic relationships of *CesA* genes based on comparison of the tunicate GH6 domain. Because the GH6 domain is found only in *Streptomyces* (as an outgroup) and tunicates in our BLAST searches, the tree consists of only the two groups. Interestingly, the tree is composed of two major groupings of branches (upper and lower groups in Figure 3). The lower group shows the tunicate *CesA* GH6 domain tree, including 11 species for which data are available. In this tree, *Oikopleura* diverged first, leaving two major, independent clades, one being the *Ciona*/*Salpa* clade and the other being the *Halocynthia*/*Botrylloides*/*Botryllus*/*Molgula* clade. This GH6-domain-based tree topology differs from that of the CesA-domain-based tree topology shown in Figure 2. As discussed above, the phylogenetic position of *Oikopleura* (larvaceans) is not consistent with the result obtained by CesA domain analysis. We favor the topology shown for the GH6 domain (Figure 3) over that for the CesA domain (Figure 2), because the branch length of the *Oikopleura* GH6 domain was comparable to those of other tunicates. This is supported by a recent report of tunicate phylogenetic relationships constructed using many nuclear genes [31].

As mentioned above, a novel finding of the *CesA* GH6 domain analysis was the presence of another *GH6* gene clade (Figure 3, upper grouping). This clade included *Oikopleura* (this branch appeared as an outgroup of the *CesA* GH6 domain), *Salpa*, *Ciona*, *Molgula*, *Botryllus*, and *Botrylloides*. This suggests that all tunicates contain two independent types of GH6 domain genes, one for the *CesA* GH6 domain and another for an independent *GH6* gene. This new finding provides additional insight into the scenario for the origin and evolution of the tunicate *CesA* gene. Tunicate *CesA* encodes a fusion protein of the CesA domain and the GH6 domain (Figure 1). As shown in our previous study [9], two *Streptomyces* genes, BLAST hits in CesA (CAB65566 in Figure 2) and GH6 (CAB65568 in Figure 3) domain analyses, are tandemly located in the genome. Given these results, we previously thought that the prokaryote *CesA-GH6* region was transferred into the genome of a tunicate ancestor and then later simply fused to form a single and large *CesA* gene. However, the presence of another *GH6* gene in tunicate genomes suggests several scenarios. Namely, one scenario suggests another horizontal transfer of the *GH6* gene after jumping of the *CesA-GH6* region into the ancestral tunicate genome. Alternatively, the *CesA-GH6* region was duplicated, and only the *CesA* region of one counterpart disappeared, leaving a *CesA-GH6* fusion gene and another *GH6* copy. *CesA* and *GH6* independently were transferred into the tunicate ancestor genome and the *GH6* was duplicated, one of which fused with *CesA* to form a unique *CesA-GH6*. Thus, under parsimonious explanation, we hypothesize that the ancestral tunicate that received *CesA* and *GH6* genes from different hosts followed a different fate. While *GH6* underwent duplication (tentatively called *GH6-1* and *GH6-2* genes), *CesA* eventually formed a fusion gene with a copy of *GH6* (*CesA*/*GH6-2*). If this is the case, what is the function of the *GH6-1* gene, and how is the expression of this gene controlled? The present result, therefore, introduces additional questions regarding uniquely horizontally-transferred genes, which unambiguously characterize one taxon of metazoans [13,33].

The ORTHOSCOPE tool makes use of sequence similarity and gene trees to find orthologs. On the other hand, a protein domain scan might be carried out in the future in order to get a more comprehensive understanding of all possible cellulose biosynthetic enzymes or cellulase enzymes in metazoans. In addition to gene models, analyses of whole genome sequences may confirm the presence or absence of the *CesA* gene. Such various analyses also find other tunicates, which use different variants of cellulose synthesizing enzymes when their genomes are sequenced.

## 4. Conclusions

In the present study using ORTHOSCOPE, we demonstrated that *CesA* is present in all available tunicate genomes. In contrast, no *CesA* orthologs were detected in genomes or transcriptomes of any metazoans examined. Therefore, the presence of *CesA* is unique to tunicates, supporting the proposal that this animal group should be recognized as a discrete animal taxon [13,34], although many questions remain to be answered regarding the origin and evolution of *CesA* in this animal group.

## Figures and Tables

**Table 1:** Number of related genes/orthologs of tunicate *CesA* CesA and GT6 domains found in the ORTHOSCOPE database.

		# of BLAST Hits ^1^		# of Related Genes/Orthologs ^1^	
Classification	Species	CesA	GH6	CesA	GH6
Bacteria	*Streptomyces coelicolor*	3	1	3	1
Viridiplantae	*Arabidopsis thaliana*	5	0	5	0
Fungi	*Saccharomyces cerevisiae*	0	0	0	0
Ichthyosporea	*Capsaspora owczarzaki*	0	0	0	0
Choanoflagellida	*Salpingoeca rosetta*	0	0	0	0
	*Monosiga brevicollis*	1	0	0	0
Metazoa					
Porifera	*Amphimedon queenslandica*	0	0	0	0
Placozoa	*Trichoplax adhaerens*	0	0	0	0
Cnidaria	*Thelohanellus kitauei*	0	0	0	0
	*Hydra vulgaris*	0	0	0	0
	*Acropora digitifera*	0	0	0	0
Ctenophora	*Mnemiopsis leidyi*	1	0	0	0
Bilateria					
Protostomia					
Platyhelminthes	*Schistosoma mansoni*	0	0	0	0
Annelida	*Capitella teleta*	0	0	0	0
Nemertea	*Notospermus geniculatus*	0	0	0	0
Brachiopoda	*Phoronis australis*	0	0	0	0
Cephalopoda	*Octopus bimaculoides*	1	0	0	0
Gastropoda	*Biomphalaria glabrata*	0	0	0	0
Bivalvia	*Crassostrea gigas*	0	0	0	0
	*Pinctada fucata*	0	0	0	0
	*Priapulus caudatus*	0	0	0	0
Nematoda	*Trichinella spiralis*	0	0	0	0
	*Caenorhabditis elegans*	0	0	0	0
Merostomata	*Limulus polyphemus*	0	0	0	0
Chilopoda	*Strigamia maritima*	0	0	0	0
Malacostraca	*Hyalella azteca*	0	0	0	0
Insecta	*Pediculus humanus*	0	0	0	0
	*Zootermopsis nevadensis*	0	0	0	0
	*Rhodnius prolixus*	0	0	0	0
	*Nasonia vitripennis*	0	0	0	0
	*Dendroctonus ponderosae*	0	0	0	0
	*Bombyx mori*	0	0	0	0
	*Drosophila melanogaster*	0	0	0	0
Deuterostomia					
Echinodermata	*Strongylocentrotus purpuratus*	0	0	0	0
	*Acanthaster planci*	0	0	0	0
Hemichordata	*Saccoglossus kowalevskii*	0	0	0	0
	*Ptychodera flava*	0	0	0	0
Cephalochordata	*Branchiostoma belcheri*	0	0	0	0
	*Branchiostoma floridae*	0	0	0	0
Vertebrata	*Gallus gallus*	0	0	0	0
	*Homo sapiens*	0	0	0	0
Tunicata					
Appendicularia	*Oikopleura dioica*	2	3	2	2
Thaliacea	*Salpa thompsoni*	1	3	1	1
Stolidobranchia	*Botrylloides leachii*	1	2	1	1
	*Botryllus schlosseri*	3	3	3	2
	*Molgula occidentalis*	1	4	1	3
	*Molgula oculate*	1	3	1	2
Enterogona	*Ciona savignyi*	1	1	1	1
	*Ciona intestinalis*	1	2	1	1

^1^ CesA or GH6 domain sequences of the four tunicate species were used as queries for each analysis (see Figure 2 and Figure 3).

## Supplementary Materials

The following are available online at https://www.mdpi.com/2073-4425/10/4/294/s1, Figure S1. Amino acid sequence alignment of tunicate GH6 and CesA proteins. Color coding follows Figure 2. Figure S2. Molecular phylogenic tree of *CesA* genes obtained using ORTHOSCOPE analysis (912 nucleotide sites). *CesA* CesA domains from three tunicates were used as query sequences (black arrows). All BLAST hits (Table 1) were used as full lengths (raw data). Therefore, in the alignment, even though the four queries consisted only of CesA domains, all BLAST hits including identical sequences, consist of full lengths. Sequences with red arrows are considered not related to the CesA domain for the following reasons: (1) The *Monosiga* sequence (XP001745167.1) was grouped with *THOC7* in an ORTHOSCOPE analysis (Figure S3). (2) In comparison with the four queries (2487–3072 bp), the *Octopus* (Ocbimv22016613m, 12,939 bp) and *Mnemiopsis* (ML10106a-RA, 9231 bp) sequences are extremely long perhaps because of mis-assemblies. See Section 3.1 for a discussion of their long branches. Figure S3. Molecular phylogenic tree obtained with an ORTHOSCOPE analysis (378 nucleotide sites) with the *Monosiga* sequence (XP001745167.1) as a query. The tree topology indicates that the *Monosiga* sequence (arrow) is related to *THOC7* but not to CesA domains. The sequence was included among the CesA domain BLAST hits (Table 1) despite the low BLAST identity score (33/119, 28%) with the *O. dioica CesA1* gene sequence (GSOIDT00017000001, shown in green). In this gene tree, because of the low identity score, the *O. dioca CesA1* gene has been included and resulted in a long branch length. Similar gene trees for the *Octopus* and *Mnemiopsis* sequences were unable to estimate, because BLAST searches using these long sequences as queries collected sequences with enormously different lengths.
