# Supplementary material for: ORTHOSCOPE Analysis Reveals the Presence of the Cellulose Synthase Gene in All Tunicate Genomes but Not in Other Animal Genomes
Source: Genes (Basel). 2019 Apr 10;10(4):294. doi: 10.3390/genes10040294 (PMC6523144; doi:10.3390/genes10040294)
Supplement: Supplementary file 1 [file genes-10-00294-s001.pdf]

Amino acid alignment of tunicate GH6 and CesA proteins

```
Oikopleura-dioica_GSOIDT00010490001_GH6-gene  ------------------------------------------------------------------------------------------------------------------------
Salpa-thompsoni_gbGFCC01117283.1.p1_GH6-gene  ------------------------------------------------------------------------------------------------------------------------
Salpa-thompsoni_gbGFCC01119318.1.p1_GH6-gene  ------------------------------------------------------------------------------------------------------------------------
Botryllus-schlosseri_botctg010729g9326_GH6-gene  ------------------------------------------------------------------------------------------------------------------------
Botrylloides-leachii_v3.S133.g02304.01.p_GH6-gene  ------------------------------------------------------------------------------------------------------------------------
Molgula-oculata_S112948.g12660.01.p_GH6-gene  ------------------------------------------------------------------------------------------------------------------------
Molgula-occidentalis_S285391.g07021.01.p_GH6-gene  ------------------------------------------------------------------------------------------------------------------------
Ciona-intestinalis_ENSCINT00000032307.1_GH6-gene  ------------------------------------------------------------------------------------------------------------------------
Oikopleura-dioica_AB543594.1_Od-CesA1-gene  ------------------------------------------------------------------------------------------------------------------------
Oikopleura-dioica_AB543593.1Od-CesA2-gene  ------------------------------------------------------------------------------------------------------------------------
Oikopleura-longicauda_AB543515.1_Ol-CesA2-gene  ------------------------------------------------------------------------------------------------------------------------
Salpa-thompsoni_gbGFCC01072613.1.p1_CesA-gene  ------------------------------------------------------------------------------------------------------------------------
Halocynthia-roretzi_AB543517.1_Hr-CesA-gene  ------------------------------------------------------------------------------------------------------------------------
Botrylloides-leachii_v3.S157.g03251.01.p_CesA-gene  ------------------------------------------------------------------------------------------------------------------------
Botryllus-schlosseri_008441g44194_CesA-gene  ------------------------------------------------------------------------------------------------------------------------
Botryllus-schlosseri_008441g44193_CesA-gene  -----------------------------------------MHESLV-------------------------------------------------------------------------
Botryllus-schlosseri_008489g44332_CesA-gene  -----------------------------------------MHESLV-------------------------------------------------------------------------
Botryllus-schlosseri_008489g44331_CesA-gene  ------------------------------------------------------------------------------------------------------------------------
Botryllus-schlosseri_008778g45080_CesA-gene  ------------------------------------------------------------------------------------------------------------------------
Molgula-oculata_S71617.g04842.01.p_CesA-gene  MDLAAFYSYSPETLDFNAFNKKHLDKQCEDSREPSQAMAVVMSLIVAIGLFGNGIVFFVILVLKEYKKSVAHWYVLQLAIADTLFLLMLPFTAHSEAVGEWSLPVGLCKAKEGILFVNFY
Molgula-oculata_v1-2.S69739.g04625.01.p_CesA-gene  ------------------------------------------------------------------------------------------------------------------------
Molgula-tectiformis_AB543516.1_Mt-CesA-gene  -----------------------------------------MTII---------------------------------------------------------------------------
Molgula-occidentalis_S469068.g15915.01.p_CesA-gene  ------------------------------------------------------------------------------------------------------------------------
Molgula-occidentalis_S469068.g15913.01.p_CesA-gene  ------------------------------------------------------------------------------------------------------------------------
Molgula-occidentalis_S469068.g15914.01.p_CesA-gene  ------------------------------------------------------------------------------------------------------------------------
Ciona-savignyi_ENSCSAVT00000016381.1_CesA-gene  -----------------------------------------MSR----------------------------------------------------------------------------
Ciona-intestinalis_BAD10864_CiCesA-gene  -----------------------------------------MSRP---------------------------------------------------------------------------

Oikopleura-dioica_GSOIDT00010490001_GH6-gene  ------------------------------------------------------------------------------------------------------------------------
Salpa-thompsoni_gbGFCC01117283.1.p1_GH6-gene  ------------------------------------------------------------------------------------------------------------------------
Salpa-thompsoni_gbGFCC01119318.1.p1_GH6-gene  ------------------------------------------------------------------------------------------------------------------------
Botryllus-schlosseri_botctg010729g9326_GH6-gene  ------------------------------------------------------------------------------------------------------------------------
Botrylloides-leachii_v3.S133.g02304.01.p_GH6-gene  ------------------------------------------------------------------------------------------------------------------------
Molgula-oculata_S112948.g12660.01.p_GH6-gene  ------------------------------------------------------------------------------------------------------------------------
Molgula-occidentalis_S285391.g07021.01.p_GH6-gene  ------------------------------------------------------------------------------------------------------------------------
Ciona-intestinalis_ENSCINT00000032307.1_GH6-gene  ------------------------------------------------------------------------------------------------------------------------
Oikopleura-dioica_AB543594.1_Od-CesA1-gene  ------------------------------------------------------------------------------------------------------------------------
Oikopleura-dioica_AB543593.1Od-CesA2-gene  ------------------------------------------------------------------------------------------------------------------------
Oikopleura-longicauda_AB543515.1_Ol-CesA2-gene  ------------------------------------------------------------------------------------------------------------------------
Salpa-thompsoni_gbGFCC01072613.1.p1_CesA-gene  ------------------------------------------------------------------------------------------------------------------------
Halocynthia-roretzi_AB543517.1_Hr-CesA-gene  ------------------------------------------------------------------------------------------------------------------------
Botrylloides-leachii_v3.S157.g03251.01.p_CesA-gene  ------------------------------------------------------------------------------------------------------------------------
Botryllus-schlosseri_008441g44194_CesA-gene  ------------------------------------------------------------------------------------------------------------------------
Botryllus-schlosseri_008441g44193_CesA-gene  -----------SR-------------QS--------------------------------------------------------------------------------------------
Botryllus-schlosseri_008489g44332_CesA-gene  -----------SR-------------QS--------------------------------------------------------------------------------------------
Botryllus-schlosseri_008489g44331_CesA-gene  ------------------------------------------------------------------------------------------------------------------------
Botryllus-schlosseri_008778g45080_CesA-gene  ------------------------------------------------------------------------------------------------------------------------
Molgula-oculata_S71617.g04842.01.p_CesA-gene  ASIAFLTIMSFDRYLAVTRIAGTGWAQEVRKMPFAMLISLMTWCGCIALATPLYRFSTVGECKRCIYSFPSSDWEECSNAGVNETACEEFLSSASNHTEDDDYYENAIDDLAKIDFGNYF
Molgula-oculata_v1-2.S69739.g04625.01.p_CesA-gene  ------------------------------------------------------------------------------------------------------------------------
Molgula-tectiformis_AB543516.1_Mt-CesA-gene  ------------------------------------------------------------------------------------------------------------------------
Molgula-occidentalis_S469068.g15915.01.p_CesA-gene  ------------------------------------------------------------------------------------------------------------------------
Molgula-occidentalis_S469068.g15913.01.p_CesA-gene  ------------------------------------------------------------------------------------------------------------------------
Molgula-occidentalis_S469068.g15914.01.p_CesA-gene  ------------------------------------------------------------------------------------------------------------------------
Ciona-savignyi_ENSCSAVT00000016381.1_CesA-gene  -----------DR-----------------------------------------------------------------------------------------------------------
Ciona-intestinalis_BAD10864_CiCesA-gene  -----------DR-----------------------------------------------------------------------------------------------------------

Oikopleura-dioica_GSOIDT00010490001_GH6-gene  ------------------------------------------------------------------------------------------------------------------------
Salpa-thompsoni_gbGFCC01117283.1.p1_GH6-gene  ------------------------------------------------------------------------------------------------------------------------
Salpa-thompsoni_gbGFCC01119318.1.p1_GH6-gene  ------------------------------------------------------------------------------------------------------------------------
Botryllus-schlosseri_botctg010729g9326_GH6-gene  ------------------------------------------------------------------------------------------------------------------------
Botrylloides-leachii_v3.S133.g02304.01.p_GH6-gene  ------------------------------------------------------------------------------------------------------------------------
Molgula-oculata_S112948.g12660.01.p_GH6-gene  ------------------------------------------------------------------------------------------------------------------------
Molgula-occidentalis_S285391.g07021.01.p_GH6-gene  ------------------------------------------------------------------------------------------------------------------------
Ciona-intestinalis_ENSCINT00000032307.1_GH6-gene  ------------------------------------------------------------------------------------------------------------------------
Oikopleura-dioica_AB543594.1_Od-CesA1-gene  --------------------------------------------------------------------MRR-------------------------------------------------
Oikopleura-dioica_AB543593.1Od-CesA2-gene  ------------------------------------------------------------------------------------------------------------------------
Oikopleura-longicauda_AB543515.1_Ol-CesA2-gene  ------------------------------------------------------------------------------------------------------------------------
Salpa-thompsoni_gbGFCC01072613.1.p1_CesA-gene  ------------------------------------------------------------------------------------------------------------------------
Halocynthia-roretzi_AB543517.1_Hr-CesA-gene  ------------------------------------------------------------------------------------------------------------------------
Botrylloides-leachii_v3.S157.g03251.01.p_CesA-gene  ----------SSVP------------------------------------------------------Y.----------------------------------SESGL-----------
Botryllus-schlosseri_008441g44194_CesA-gene  ------------------------------------------------------------------------------------------------------------------------
Botryllus-schlosseri_008441g44193_CesA-gene  -----------------------------------------------GSAERT---------------I..-------------------------------------------------
Botryllus-schlosseri_008489g44332_CesA-gene  -----------------------------------------------GSAERT---------------I..-------------------------------------------------
Botryllus-schlosseri_008489g44331_CesA-gene  ------------------------------------------------------------------------------------------------------------------------
Botryllus-schlosseri_008778g45080_CesA-gene  ------------------------------------------------------------------------------------------------------------------------
Molgula-oculata_S71617.g04842.01.p_CesA-gene  LGDFEDFFENDPTPICRDEPLNSYKLWIWLNFVIAFLLPLIAICICYGLILKTMMSTGTLVQNSNQRRY..RVTFMVLALVSLFIISWLPWYVVKLVKLKGFEMSASGCKVLNQFTLVLA
Molgula-oculata_v1-2.S69739.g04625.01.p_CesA-gene  ------------------------------------------------------------------------------------------------------------------------
Molgula-tectiformis_AB543516.1_Mt-CesA-gene  ----------DMTP-------SNLKIWI-------------------------------LVVDHEQ----------CLA-ADILVIA------------------------------VLC
Molgula-occidentalis_S469068.g15915.01.p_CesA-gene  ------------------------------------------------------------------------------------------------------------------------
Molgula-occidentalis_S469068.g15913.01.p_CesA-gene  ------------------------------------------------------------------------------------------------------------------------
Molgula-occidentalis_S469068.g15914.01.p_CesA-gene  ------------------------------------------------------------------------------------------------------------------------
Ciona-savignyi_ENSCSAVT00000016381.1_CesA-gene  ---------RHELP---------------------------------GA-------------------Y..-------------------------------SEQPGGR-----------
Ciona-intestinalis_BAD10864_CiCesA-gene  ---------NQDLP---------------------------------GA-------------------Y..-------------------------------SGFSEGR-----------

Oikopleura-dioica_GSOIDT00010490001_GH6-gene  ------------------------------------------------------------------------------------------------------------------------
Salpa-thompsoni_gbGFCC01117283.1.p1_GH6-gene  ------------------------------------------------------------------------------------------------------------------------
Salpa-thompsoni_gbGFCC01119318.1.p1_GH6-gene  ------------------------------------------------------------------------------------------------------------------------
Botryllus-schlosseri_botctg010729g9326_GH6-gene  ------------------------------------------------------------------------------------------------------------------------
Botrylloides-leachii_v3.S133.g02304.01.p_GH6-gene  ------------------------------------------------------------------------------------------------------------------------
Molgula-oculata_S112948.g12660.01.p_GH6-gene  ------------------------------------------------------------------------------------------------------------------------
Molgula-occidentalis_S285391.g07021.01.p_GH6-gene  ------------------------------------------------------------------------------------------------------------------------
Ciona-intestinalis_ENSCINT00000032307.1_GH6-gene  ------------------------------------------------------------------------------------------------------------------------
Oikopleura-dioica_AB543594.1_Od-CesA1-gene  ------------------------------------------------------------------------------------------------------------------------
Oikopleura-dioica_AB543593.1Od-CesA2-gene  ------------------------------------------------------------------------------------------------------------------------
Oikopleura-longicauda_AB543515.1_Ol-CesA2-gene  ------------------------------------------------------------------------------------------------------------------------
Salpa-thompsoni_gbGFCC01072613.1.p1_CesA-gene  ------------------------------------------------------------------------------------------------------------------------
Halocynthia-roretzi_AB543517.1_Hr-CesA-gene  ------------------------------------------------------------------------------------------------------------------------
Botrylloides-leachii_v3.S157.g03251.01.p_CesA-gene  ---------------------------TSDRSHT------------------------------------------------------------APRHPSSRYI-SSATTNADQDFHNGS
Botryllus-schlosseri_008441g44194_CesA-gene  ------------------------------------------------------------------------------------------------------------------------
Botryllus-schlosseri_008441g44193_CesA-gene  ------------------------FSNEMNRSIE--------------------------------------------------------------------------------------
Botryllus-schlosseri_008489g44332_CesA-gene  ------------------------FSNEMNRSIE--------------------------------------------------------------------------------------
Botryllus-schlosseri_008489g44331_CesA-gene  ------------------------------------------------------------------------------------------------------------------------
Botryllus-schlosseri_008778g45080_CesA-gene  ------------------------------------------------------------------------------------------------------------------------
Molgula-oculata_S71617.g04842.01.p_CesA-gene  YLNSALNPYFYSFMGSHFYRRFERAKSTAQRSKLLSALSRMSSHKADSSNSQSERNGSTSNARKNSKKTFLRQDSESKGLGAYKRSTMSSQQSRDPRY-SSRHS-SSTNPNGDEYRHDSF
Molgula-oculata_v1-2.S69739.g04625.01.p_CesA-gene  ------------------------------------------------------------------------------------------------------------------------
Molgula-tectiformis_AB543516.1_Mt-CesA-gene  HL----------------------------RVWV------------------------------------------------------------------------------EETREE--
Molgula-occidentalis_S469068.g15915.01.p_CesA-gene  ------------------------------RSR----------------------------------------------------------------------K-STTLPN---QRYD--
Molgula-occidentalis_S469068.g15913.01.p_CesA-gene  ------------------------------------------------------------------------------------------------------------------------
Molgula-occidentalis_S469068.g15914.01.p_CesA-gene  ------------------------------------------------------------------------------------------------------------------------
Ciona-savignyi_ENSCSAVT00000016381.1_CesA-gene  ---------------------------SLPRSSI------------------------------------------------------------PPK--PSRYTSSSFATSENDFGPDHF
Ciona-intestinalis_BAD10864_CiCesA-gene  ---------------------------SLPRSSI------------------------------------------------------------PPK--PSRYT-SSYGTSENDFGPDHF
```

```
Oikopleura-dioica_GSOIDT00010490001_GH6-gene ------------------------------------------------------------------------------------------------------------------------
Salpa-thompsoni_gbGFCC01117283.1.p1_GH6-gene ------------------------------------------------------------------------------------------------------------------------
Salpa-thompsoni_gbGFCC01119318.1.p1_GH6-gene ------------------------------------------------------------------------------------------------------------------------
Botryllus-schlosseri_botctg010729g9326_GH6-gene ------------------------------------------------------------------------------------------------------------------------
Botrylloides-leachii_v3.S133.g02304.01.p_GH6-gene ------------------------------------------------------------------------------------------------------------------------
Molgula-oculata_S112948.g12660.01.p_GH6-gene ------------------------------------------------------------------------------------------------------------------------
Molgula-occidentalis_S285391.g07021.01.p_GH6-gene ------------------------------------------------------------------------------------------------------------------------
Ciona-intestinalis_ENSCINT00000032307.1_GH6-gene ------------------------------------------------------------------------------------------------------------------------
Oikopleura-dioica_AB543594.1_Od-CesA1-gene ------------------------------------------------------------------------------------------------------------------------
Oikopleura-dioica_AB543593.10d-CesA2-gene ------------------------------------------------------------------------------------------------------------------------
Oikopleura-longicauda_AB543515.1_Ol-CesA2-gene ------------------------------------------------------------------------------------------------------------------------
Salpa-thompsoni_gbGFCC01072613.1.p1_CesA-gene ------------------------------------------------------------------------------------------------------------------------
Halocynthia-roretzi_AB543517.1_Hr-CesA-gene ------------------------------------------------------------------------------------------------------------------------
Botrylloides-leachii_v3.S157.g03251.01.p_CesA-gene YSGDTDE----HSRSNRTMPARR--YRSGYSEDPS-----------TRSSSNSLSSNGKRPSQNTTTGSDXTQI-----SEDSSRTND----WHQRRG-DDSSSYTSAESYDTDDTE---
Botryllus-schlosseri_008441g44194_CesA-gene ------------------------------------------------------------------------------------------------------------------------
Botryllus-schlosseri_008441g44193_CesA-gene ---------------------------------------------------------------------------------------------------------------DTDDTE---
Botryllus-schlosseri_008489g44332_CesA-gene ---------------------------------------------------------------------------------------------------------------DTDDTE---
Botryllus-schlosseri_008489g44331_CesA-gene ------------------------------------------------------------------------------------------------------------------------
Botryllus-schlosseri_008778g45080_CesA-gene ------------------------------------------------------------------------------------------------------------------------
Molgula-oculata_S71617.g04842.01.p_CesA-gene NSEDMDI----GSRSRNTLPSR-------YSRDSQ-----------SDMSTGSLDRRHQRRGN-----GDSNFT-----ISDTSGAR-------------SQASYS---SYDSDDTE-TD
Molgula-oculata_v1-2.S69739.g04625.01.p_CesA-gene ------------------------------------------------------------------------------------------------------------------------
Molgula-tectiformis_AB543516.1_Mt-CesA-gene -----------------------------------------------VMPTQVLQK--------------------------APALG-------------SQTSYSSTGSYESDDTDTTD
Molgula-occidentalis_S469068.g15915.01.p_CesA-gene --------------------SR-------YSRDNG-----------SVISAATLDNRGKRRSH-----ADTSFT-----VSDSSGSR-------------SQTSYSSADSYDSDETE--D
Molgula-occidentalis_S469068.g15913.01.p_CesA-gene ------------------------------------------------------------------------------------------------------------------------
Molgula-occidentalis_S469068.g15914.01.p_CesA-gene ------------------------------------------------------------------------------------------------------------------------
Ciona-savignyi_ENSCSAVT00000016381.1_CesA-gene NFNDSDTSAS-RSRVTSTLPSGQ--GSSGFSRSGSEAPPIPDNKRRTISMGANLQPPRAKRSMGGRH-RDRNQTLPPYSVSDSSRRTMSGNDENNRFGSDGSSIYSSADSFDSQDDF--D
Ciona-intestinalis_BAD10864_CiCesA-gene NFTDGDTSASGRSRNASTLPSGPSGSSAAFSIRDSHPPPVSDSKRRTISMGANIQPPRSKRSGGTRH-----------------------------------------------------
```

```
Oikopleura-dioica_GSOIDT00010490001_GH6-gene ------------------------------------------------------------------------------------------------------------------------
Salpa-thompsoni_gbGFCC01117283.1.p1_GH6-gene ------------------------------------------------------------------------------------------------------------------------
Salpa-thompsoni_gbGFCC01119318.1.p1_GH6-gene ------------------------------------------------------------------------------------------------------------------------
Botryllus-schlosseri_botctg010729g9326_GH6-gene ------------------------------------------------------------------------------------------------------------------------
Botrylloides-leachii_v3.S133.g02304.01.p_GH6-gene ------------------------------------------------------------------------------------------------------------------------
Molgula-oculata_S112948.g12660.01.p_GH6-gene ------------------------------------------------------------------------------------------------------------------------
Molgula-occidentalis_S285391.g07021.01.p_GH6-gene ------------------------------------------------------------------------------------------------------------------------
Ciona-intestinalis_ENSCINT00000032307.1_GH6-gene ------------------------------------------------------------------------------------------------------------------------
Oikopleura-dioica_AB543594.1_Od-CesA1-gene --------------SNSSDSFEYTTGP---------------------------------------------------------------------------------------------
Oikopleura-dioica_AB543593.10d-CesA2-gene ---------------M..GNYRG.V-----------------------------------------------------------------------------------------------
Oikopleura-longicauda_AB543515.1_Ol-CesA2-gene ------------------------------------------------------------------------------------------------------------------------
Salpa-thompsoni_gbGFCC01072613.1.p1_CesA-gene ------------------------------------------------------------------------------------------------------------------------
Halocynthia-roretzi_AB543517.1_Hr-CesA-gene ------------------------------------------------------------------------------------------------------------------------
Botrylloides-leachii_v3.S157.g03251.01.p_CesA-gene --TGRRRSRKSR-DTSH.VDYGGRYY.DVMDNDEMRRLQSGYSQDTSTGTDSTITNGDAIDRRQSDMSELANYFNRRLEDMQKRQ----SRI-TSRGTGQTESYPGEVNFPENYN-RSRH
Botryllus-schlosseri_008441g44194_CesA-gene ------------------------------------------------------------------------------------------------------------------------
Botryllus-schlosseri_008441g44193_CesA-gene --TNRRRSRKSR-DASR.MDYGGRYY.DVMDNDEMRRLQSGYSQD-STGTDTTITNGDGVSARQSDMNELANYFNRRLEDMQKRQ----SRI--ESRGGQGDNYPGDLNSVNSSS-RDRY
Botryllus-schlosseri_008489g44332_CesA-gene --TNRRRSRKSR-DASR.MDYGGRYY.DVMDNDEMRRLQSGYSQD-STGTDTTITNGDGVSARQSDMNELANYFNRRLEDMQKRQ----SRI--ESRGGQGDNYPGDLNSVNSSS-RDRY
Botryllus-schlosseri_008489g44331_CesA-gene ------------------------------------------------------------------------------------------------------------------------
Botryllus-schlosseri_008778g45080_CesA-gene ------------------------------------------------------------------------------------------------------------------------
Molgula-oculata_S71617.g04842.01.p_CesA-gene YRSGRHRSKNT---ES.FSNYTGRYY.DPMES--MNGLQSNISKETAT-TDTTLTNNDI---KGKNIDELADYFNRRLEDMQRQA----SQIGDSSRRR-RNDYPDDLTSNYSYQ-RGGT
Molgula-oculata_v1-2.S69739.g04625.01.p_CesA-gene ------------------------------------------------------------------------------------------------------------------------
Molgula-tectiformis_AB543516.1_Mt-CesA-gene YRNGRHRSKNT---ES.ASNYTGRFY.DPIES--MNGLQSNISKDTAT-TDTTLTNNDI---KGKNIDELADYFNKRLEDMQRQA----SQM-EGPRRRNRSQYPDDLTSNYSYQHRDET
Molgula-occidentalis_S469068.g15915.01.p_CesA-gene YRSDRGRSKN----QS.VSNYTGRYY.DPMES--MNRLHSDASKVTAT-TDTTLTNNDI---RQKSIDELADYFNRRLEDMQRQA----SQIDNGPRRRNRDQYPDDLTSNYSYQ-RGET
Molgula-occidentalis_S469068.g15913.01.p_CesA-gene ------------------------------------------------------------------------------------------------------------------------
Molgula-occidentalis_S469068.g15914.01.p_CesA-gene ------------------------------------------------------------------------------------------------------------------------
Ciona-savignyi_ENSCSAVT00000016381.1_CesA-gene RRFPPRPPKPDKYK.S..SN.TGQFY.DPMQSADLGRLQSGYSED----TNTTITNGNS---RPTNLDELAEYFNKKLEDVQRQADSAMNRMQNGRNRSQRSESTQD-SSVFSYP-SGPS
Ciona-intestinalis_BAD10864_CiCesA-gene ------------------------------------------------------------------------------------------------------------------------
```

```
Oikopleura-dioica_GSOIDT00010490001_GH6-gene -----------------------------------------------MDAKHAA.-----------------------------------------------------------------
Salpa-thompsoni_gbGFCC01117283.1.p1_GH6-gene ------------------------------------------------------------------------------------------------------------------------
Salpa-thompsoni_gbGFCC01119318.1.p1_GH6-gene ------------------------------------------------------------------------------------------------------------------------
Botryllus-schlosseri_botctg010729g9326_GH6-gene ------------------------------------------------------------------------------------------------------------------------
Botrylloides-leachii_v3.S133.g02304.01.p_GH6-gene ------------------------------------------------------------------------------------------------------------------------
Molgula-oculata_S112948.g12660.01.p_GH6-gene ------------------------------------------------------------------------------------------------------------------------
Molgula-occidentalis_S285391.g07021.01.p_GH6-gene ------------------------------------------------------------------------------------------------------------------------
Ciona-intestinalis_ENSCINT00000032307.1_GH6-gene ------------------------------------------------------------------------------------------------------------------------
Oikopleura-dioica_AB543594.1_Od-CesA1-gene ------------SHRPSNRP-EREKTYESIEPNYRDRPPRPSKPPSITTTNRSKTNHNVHSWSRTNNSAPPS-NASSFSRPDHARAHDELPSRPSTLNIRSEISRIRAELRQLELEESK
Oikopleura-dioica_AB543593.10d-CesA2-gene ---------------..TN.A-VSQEG---------------QG..EGDRVNG.L--.NIR---------------------------..IA.------L..Q...GIET.GSTRPGQDS
Oikopleura-longicauda_AB543515.1_Ol-CesA2-gene -------------------------------------------M.MAGSSIGG.Q--.NL------------------------------------------------------------
Salpa-thompsoni_gbGFCC01072613.1.p1_CesA-gene --------------M...N.A-MSGF----------------.QDG.M.P.NSRL--TSS-------K.LVTE---.GLT-----AVR..IEN------LKIQ.K.GLTHFGENGESSMQ
Halocynthia-roretzi_AB543517.1_Hr-CesA-gene ------------------------------------------------------------------------------------------------------------------------
Botrylloides-leachii_v3.S157.g03251.01.p_CesA-gene DPLEY----NGR-TI..TN.A-ISG------------------QTSEM.P.NS-------------------------------------------------------------------
Botryllus-schlosseri_008441g44194_CesA-gene ------------------------------------------------------------------------------------------------------------------------
Botryllus-schlosseri_008441g44193_CesA-gene DTSGF----NNR-TV..TN.A-ISS------------------QISDM.P.NSHT------------------------------AYE..------------------------------
Botryllus-schlosseri_008489g44332_CesA-gene DTSGF----NNR-TV..TN.A-ISS------------------QISDM.P.NSHT------------------------------AYEX.------------------------------
Botryllus-schlosseri_008489g44331_CesA-gene ------------------------------------------------------------------------------------------------------------------------
Botryllus-schlosseri_008778g45080_CesA-gene ------------------------------------------------------------------------------------------------------------------------
Molgula-oculata_S71617.g04842.01.p_CesA-gene DGDSVRSFDHGR-TI..TN.A-ISG-------------------LSE..P.NSEN--------------LTTERSG.RI.-----SVQ..IET------L..Q...DLDTKSSDRNT.D-
Molgula-oculata_v1-2.S69739.g04625.01.p_CesA-gene ------------------------------------------------------------------------------------------------------------------------
Molgula-tectiformis_AB543516.1_Mt-CesA-gene DEDSVRSFDHGR-TI..TN.A-ISG-------------------MSDM.P.NSEN--.----------MM.TE-SG.RI.-----SVQ..IET------L..Q...DLDSKSSGRNT.D-
Molgula-occidentalis_S469068.g15915.01.p_CesA-gene DDDSVRSFNHGR-TI..TN.A-MSG-------------------MSDM.P.NSEN--.----------MM.TE-SG.RI.-----SVQ..IET------L..Q...DLDSKSSDKNT.D-
Molgula-occidentalis_S469068.g15913.01.p_CesA-gene ------------------------------------------------------------------------------------------------------------------------
Molgula-occidentalis_S469068.g15914.01.p_CesA-gene ------------------------------------------------------------------------------------------------------------------------
Ciona-savignyi_ENSCSAVT00000016381.1_CesA-gene ---------GGRHTI..TN.AGISS------------------DAST..P.NSRS--YQTM-----ES.Q.TE---..I.-----AVR..IE.------LK.QL..GLDNMTSRGESQDM
Ciona-intestinalis_BAD10864_CiCesA-gene ------------------------------------------------------------------------------------------------------------------------
```

```
Oikopleura-dioica_GSOIDT00010490001_GH6-gene ---------------------------------------------------------------------------------AQEAGLS--------------------------------
Salpa-thompsoni_gbGFCC01117283.1.p1_GH6-gene ------------------------------------------------------------------------------------------------------------------------
Salpa-thompsoni_gbGFCC01119318.1.p1_GH6-gene ------------------------------------------------------------------------------------------------------------------------
Botryllus-schlosseri_botctg010729g9326_GH6-gene -------------------------------------------------------------------MSWEAR------------SIG--------------------------------
Botrylloides-leachii_v3.S133.g02304.01.p_GH6-gene ------------------------------------------------------------------------------------------------------------------------
Molgula-oculata_S112948.g12660.01.p_GH6-gene ------------------------------------------------------------------------------------------------------------------------
Molgula-occidentalis_S285391.g07021.01.p_GH6-gene ------------------------------------------------------------------------------------------------------------------------
Ciona-intestinalis_ENSCINT00000032307.1_GH6-gene ------------------------------------------------------------------------------------------------------------------------
Oikopleura-dioica_AB543594.1_Od-CesA1-gene SPSEMAELTSLYELQLRKMI--------------------------HEQVPED----------------------------SSIHEFIHPE----DTIRPPSRPVKNRAPPPPEQAPSVS
Oikopleura-dioica_AB543593.10d-CesA2-gene .ARD.E..ADYFSQR.A.CQKQVYNVVNHVHSEV--------DQGG.Q.D.YNPNARYASGKG---GKNWD----ENGDFD..DSGIGQ.KPGARG....AG.AAHRGG..-----S.IG
Oikopleura-longicauda_AB543515.1_Ol-CesA2-gene ----------------------------------------------AQRD.YN-------------------------------------------------------------------
Salpa-thompsoni_gbGFCC01072613.1.p1_CesA-gene Y---.DQMATFFSKR.EDCTKQVTDAVSKVQSQVGEIKRSY--------E.RN--GRNISKRGFAPPQEFAGRAGNEMYYD..DSGIG-.K---GL.V..QT.ARSDLKAT--.SNTTEQ
Halocynthia-roretzi_AB543517.1_Hr-CesA-gene ------------------------------------------------------------------------------------------------------------------------
Botrylloides-leachii_v3.S157.g03251.01.p_CesA-gene --------------------QMVYDQENSSQSRVGQVRESMNGA------------RR--------ASEWESR--EEESYD..DSGIG-.R---NL....MT.AHSDALSE--R.PSTLR
Botryllus-schlosseri_008441g44194_CesA-gene --------------------------------------------------------------------------------------MX-.R-----------------------------
Botryllus-schlosseri_008441g44193_CesA-gene ---------------------------NTSQSRVGPDRE-------NAGA.RM-------------QGEWESR--EEEMYD..DSGIG-.R---NL....MT.AHANALAN--R.PS..-
Botryllus-schlosseri_008489g44332_CesA-gene ---------------------------NTSQSRVGPDRE-------NAGA.RM-------------QGEWESR--EEEMYD..DSGIG-.R---NL....MT.AHANALAN--R.PS..-
Botryllus-schlosseri_008489g44331_CesA-gene ------------------------------------------------------------------------------------------------------------------------
Botryllus-schlosseri_008778g45080_CesA-gene ------------------------------------------------------------------------------------------------------------------------
Molgula-oculata_S71617.g04842.01.p_CesA-gene -------.ANYFSKR.EECQHRVYSAVNNVQSHVGHVRSSVADSSVQSSR.R---HRR--------RQDYYGR--EDDYDD..DSGIG-.R---NL....MT.AQARE.QE--RA.S---
Molgula-oculata_v1-2.S69739.g04625.01.p_CesA-gene ------------------------------------------------------------------------------------------------------------------------
Molgula-tectiformis_AB543516.1_Mt-CesA-gene -------.ANYFSKR.EECQQQVYSAVNNVQSHVGHVRSSVADSSVQSSR.R---HRR--------RHDYNGR--DDDYDD..DSGIG-.R---NL....MT.AQARE.QE--RAGS---
Molgula-occidentalis_S469068.g15915.01.p_CesA-gene -------.ANYFSRR.EECQQQVYNAVNNVQSHVGHVRSSVADSSV.SSR.R---HRR--------RHDYSGR--DDD-GD..DSGIG-.R---NL....MT.AQARE.QE--RA.S---
Molgula-occidentalis_S469068.g15913.01.p_CesA-gene ------------------------------------------------------------------------------------------------------------------------
Molgula-occidentalis_S469068.g15914.01.p_CesA-gene ------------------------------------------------------------------------------------------------------------------------
Ciona-savignyi_ENSCSAVT00000016381.1_CesA-gene -----D.MADYFSKR.EECQNQVYKAVNQVQGQMGEPKRGF--------E.RQ--SRH--------RQDWENR--DDEFYD..DSGIG-.R---NL....LT.AHANALEN--N.P.---
Ciona-intestinalis_BAD10864_CiCesA-gene ------------------------------------------------------------------------R--DDEVYD..DSGIG-.R---NL....LT.AHANALEN--N.P.---
```

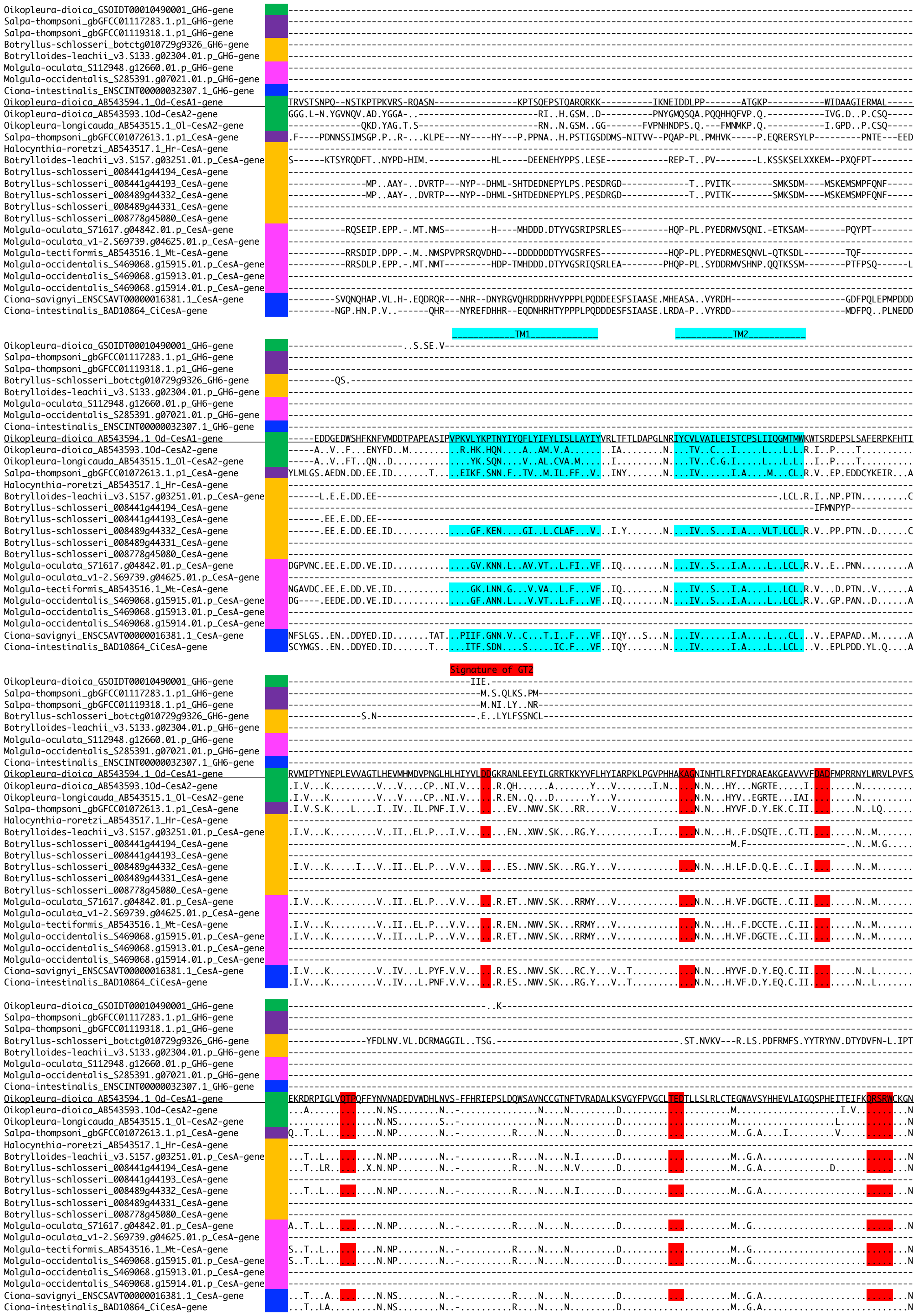

Fig. S1- 3

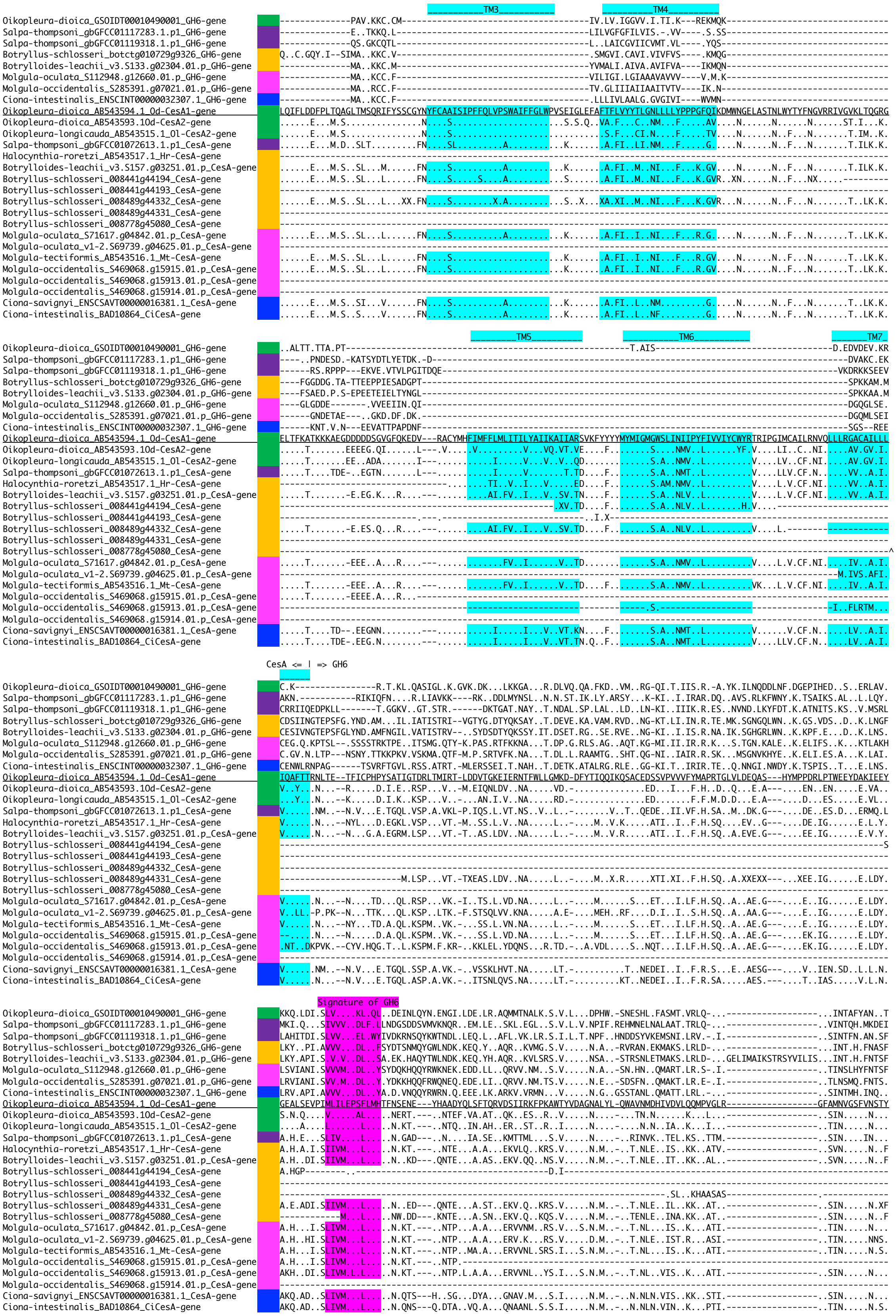

Fig. S1- 4

```
Oikopleura-dioica_GSOIDT00010490001_GH6-gene VELF.RK.S.A.N.H.IM..G.N..A..QKK.LE.AS..R....D.RKSNN..W.FA...PVDA---RGRRRR-----------------------------------------------
Salpa-thompsoni_gbGFCC01117283.1.p1_GH6-gene SEFW.RK.F.R...H.I.....N..T..-..NQNQ.MS.PF.....KP..S..WAW..RNKVV---------------------------------------------------------
Salpa-thompsoni_gbGFCC01119318.1.p1_GH6-gene TEY..RDIF.K...H.I.....NR.T..-KLPI.Q.QK.LF...G.AK.SP..WSW..RN------------------------------------------------------------
Botryllus-schlosseri_botctg010729g9326_GH6-gene TNVM.RR...L...H.IT....N..L..-RQ.ISS.Q..MF...RMLA.LP..WSW.TRSS-----------------------------------------------------------
Botrylloides-leachii_v3.S133.g02304.01.p_GH6-gene TNSM.RR...I...H.IT....N..T..-RQ.VGH.SK.FF...GMLA.LP..WSW.T.TS-----------------------------------------------------------
Molgula-oculata_S112948.g12660.01.p_GH6-gene NSRI.GR.N.K...HFLV..G.N..K..-RQHASK.IQ.L....N.KA.SS..WSW.VRSD.N---------------------------------------------------------
Molgula-occidentalis_S285391.g07021.01.p_GH6-gene NNKI.MR...K...HFIV..G.N..AY.-RQAK.Q.QK.LF...H.KA.SN..WSW.VRSEVS---------------------------------------------------------
Ciona-intestinalis_ENSCINT00000032307.1_GH6-gene NNLI..R...R...NF.V....NR.E..-TK.IL.MER.LF...HVRK.Q..SW.W...T.T----------------------------------------------------------
Oikopleura-dioica_AB543594.1_Od-CesA1-gene NMQLASELHCQTGLNYVIDTSRNGGIFS-DRSMDEINECTYDPPYISRGAIPGYGPGSKKAINR-VDTD------------------------------------------LKVDNDY--
Oikopleura-dioica_AB543593.1Od-CesA2-gene NL...T.I......NFIL....N..M..-E.TL...N.......FV.K.SE..WAG...NPVNQ-KS.GAM--------------------------------------DFGSITN.F--
Oikopleura-longicauda_AB543515.1_Ol-CesA2-gene NL...T.I......NFIL....N..L..-E..LED.N........VGK.SA..WAA..S.QVEQ-ITDG---------------------------------------------IP..--
Salpa-thompsoni_gbGFCC01072613.1.p1_CesA-gene NEMI.NSIF.E...H.L.....N..EL.-.M.L.D.N......NVKN.T..MWR....VKEISHKGGGEIL------------LAEGT--------------------------------
Halocynthia-roretzi_AB543517.1_Hr-CesA-gene NR...E.I......H.IV....N..E..-T.TLE..NL......KVEK....SWQI.AT.SAL----QGQLL------AEQMDVSASPAAPAA--------------------------
Botrylloides-leachii_v3.S157.g03251.01.p_CesA-gene NR...E.I......H.IV....S..E..-K..IE..N.......MVEK.DA.SWQT..GVKQH----QAQL-----------LASGTTLVD----------------------------
Botryllus-schlosseri_008441g44194_CesA-gene ----------------------S.R...-ILA------------------A.DWSRM.TFSLR----PKSL------------LISMTFVTE----------------------------
Botryllus-schlosseri_008441g44193_CesA-gene ------------------------------------------------------------------------------------------------------------------------
Botryllus-schlosseri_008489g44332_CesA-gene ---------------------.MC.AYF-SS.L---------------------------------------------------------------------------------------
Botryllus-schlosseri_008489g44331_CesA-gene NR...D.I......H.......N..E..-T..IE..N.......MTEK..T.SWQN.AAIQQH----QAQR-----------LANSDSLPSPD--------------------------
Botryllus-schlosseri_008778g45080_CesA-gene NR...D.I......H.......N..E..-T..IE..N.......MTEK.VA.SWQS.AAIQQY----QAHR-----------LANSDSLPSPD--------------------------
Molgula-oculata_S71617.g04842.01.p_CesA-gene NEL..E.I......H.LV....N..D..-T..L.D.N.......MVNK.N..SWMD.AQIRKL----AGQIASEAF-SGITSDTAEVPVASD---------------------------
Molgula-oculata_v1-2.S69739.g04625.01.p_CesA-gene NELI.D.IM.....H.LV....N..D..-T..L...N..E....MVNK.NT.SWMD.A.SRKV----GGQIASEVFGSG----------------------------------------
Molgula-tectiformis_AB543516.1_Mt-CesA-gene NEL..E.I......H.LV....N..D..-R..L.D.N.......EVGK.E..SWAT.AGVTLL----AGQRA----------LASIPVQTE----------------------------
Molgula-occidentalis_S469068.g15915.01.p_CesA-gene ------------------------------------------------------------------------------------------------------------------------
Molgula-occidentalis_S469068.g15913.01.p_CesA-gene NQL..E.I......H.LV....N..E..-T..L.D.N.......Q.GK.ES.HWEA.AVVQTL----AGQV-------GLNTMVQETPVSTDTDT-------------------------
Molgula-occidentalis_S469068.g15914.01.p_CesA-gene -------------------------------.L.D.N.......QVGK.ES.HWEA.AVVQTL----AGQV-------GLNTMVQETPVSTDTDT-------------------------
Ciona-savignyi_ENSCSAVT00000016381.1_CesA-gene NEI..N.I.....YH.I.....N..E..-T..L.D.NS.....RVKN.SM.MWKD..AVTTL.--TAGSN-------GGAT-AVTLPPATVVPSVKKRWVLNSPTDMGDSEYYGG..KY
Ciona-intestinalis_BAD10864_CiCesA-gene NEI...I.....YHFL.....N..E..-S..L...NS.....RVRN.SR.TWRD.AATVTL----VGQT-------GLASAAATVPP-------------------------------

Oikopleura-dioica_GSOIDT00010490001_GH6-gene ----------SSSN------N-------------------------------------AMS.S..GLYSTGLGEQQVVNRGWSSQRPLLSPYERNTLANAPMEGMPQL-------------
Salpa-thompsoni_gbGFCC01117283.1.p1_GH6-gene ----------------------------------------------------------A-------------------------------------------------------------
Salpa-thompsoni_gbGFCC01119318.1.p1_GH6-gene ------------------------------------------------------------------------------------------------------------------------
Botryllus-schlosseri_botctg010729g9326_GH6-gene ------------------------------------------------------------L.QK.W--------------SPNFDPPP---QNNHQPPPMRTSQINNPVVSVPRQPPNQQ
Botrylloides-leachii_v3.S133.g02304.01.p_GH6-gene ------------------------------------------------------------P.QK.W--------------SPNFNPP------SHSPPQQPPQQQPSYMIIPNRQPPNQQ
Molgula-oculata_S112948.g12660.01.p_GH6-gene ---------------------------------------------------------QYS.R..WVMTDT----SSSNTSPQTNPPV---RLTQSA---------PHTVWPSSRQGPRRN
Molgula-occidentalis_S285391.g07021.01.p_GH6-gene ---------------------------------------------------------Q-P.SK.WVMNVG----QGLSNRP-TSPPV---RITQSSFQRTTSRRP-----PPPRNNPNFN
Ciona-intestinalis_ENSCINT00000032307.1_GH6-gene ------------------------------------------------------------L.TK.Y------------------------------------------------------
Oikopleura-dioica_AB543594.1_Od-CesA1-gene ---------------D--------------------DYY-GDYYYSEY---YRKRRSTVPTRHRRQE------------------------------------------------------
Oikopleura-dioica_AB543593.1Od-CesA2-gene ----------GAEAY.--------------------S.S-S--S.D..A--.....E.G-..QK.WSMGD--------------------------------------------------
Oikopleura-longicauda_AB543515.1_Ol-CesA2-gene ----------SAPAS.--------------------..G-.--.GDD.AYD..R...AG--.QK.FDMNA--------------------------------------------------
Salpa-thompsoni_gbGFCC01072613.1.p1_CesA-gene ----------TAVP----------------------..E-------------------.RR.RK.WTFDE--------YGSDAGAGPV---YEAGNPAAGG-------------------
Halocynthia-roretzi_AB543517.1_Hr-CesA-gene ----------GATP---------AETEDTA------E.DL.YI---------------G--.KK.WSLE---------PVNDGTAPI---YESDNIA-----------------------
Botrylloides-leachii_v3.S157.g03251.01.p_CesA-gene ----------SSNVL--------AEEEAAANPNQPV..SL.GTFGN.-----------GKK.RK.WNFP---------GQEEG-API---FESQNPAAGG--TLP---------------
Botryllus-schlosseri_008441g44194_CesA-gene ----------X-------------------------------------------------------------------------------------------------------------
Botryllus-schlosseri_008441g44193_CesA-gene ------------------------------------------------------------------------------------------------------------------------
Botryllus-schlosseri_008489g44332_CesA-gene ------------------------------------H.K---------------------------------------------------------------------------------
Botryllus-schlosseri_008489g44331_CesA-gene ----------TSRA---------DNTEEVGTDEAP-...L.GIFGN------------SKK.RK.WNFPG--------DEPEGAGPI---YEAGNPAAGG--------------------
Botryllus-schlosseri_008778g45080_CesA-gene ----------TSRA---------GNTEVVDTDEAP-..SF.GIFGN------------SKR.RK.WNLPG--------DETEGSGPI---YEAGNPAAGG--------------------
Molgula-oculata_S71617.g04842.01.p_CesA-gene ----------TAA------------ADTSYEDAAF..S------NQAI----------G-R.KK.W.LD---------PVNIGNNPV---YSDGNLAAGGVVNKP---------------
Molgula-oculata_v1-2.S69739.g04625.01.p_CesA-gene ----------AETPD.DEG-----------------N.F------NKRI---------G-R.KK.WVLDS--------VNTVES---K---YTVDDPAVGG-------------------
Molgula-tectiformis_AB543516.1_Mt-CesA-gene ----------VEAP----------ETANTETESEYY..E------NYQI---------GKK.KK.W.LD---------APPAG-GPI---FAEDSPAAGG--QQP---------------
Molgula-occidentalis_S469068.g15915.01.p_CesA-gene ------------------------------------------------------------------------------------------------------------------------
Molgula-occidentalis_S469068.g15913.01.p_CesA-gene ----------TESEYLNYD-----------------E.QI.-----------------GKR.KK.S.LD---------VTNTGNEPV---YYSDDSAGAG--------------------
Molgula-occidentalis_S469068.g15914.01.p_CesA-gene ----------TESEYLNYD-----------------E.QI.-----------------GKR.KK.W.LD---------ATNTGNEPV---Y-SDSPAAGGVANPP---------------
Ciona-savignyi_ENSCSAVT00000016381.1_CesA-gene YYSGNIQANLTGDPLNGGKLSTGGEKSSSNASDAGLS..-----GNPKALLSQN.EEKE.RIQK.WALN---------HPAVG-SAV---VEPNNPAAGG--TLT---------------
Ciona-intestinalis_BAD10864_CiCesA-gene ----------TETPL-------------------------------PAS------------VKK.WALN---------APAAG-DPV---VEANNPAAGG--ALP---------------

Oikopleura-dioica_GSOIDT00010490001_GH6-gene ---------------KMANQFGGNGGLPSRTASG--KIRAIQKAPRPSNIGAS-GAGKK------------------------------------------------AASKG--------
Salpa-thompsoni_gbGFCC01117283.1.p1_GH6-gene -------------------------------------------------------VGSI------------------------Y.STTTTH.Q---------------------------
Salpa-thompsoni_gbGFCC01119318.1.p1_GH6-gene -----------------------------------------------------------------------------------------------.S.TERIE----GV-----------
Botryllus-schlosseri_botctg010729g9326_GH6-gene ENPE--------EYNRWMNEMRNGGG--QNSGYA--QAGSVPQHQPQN.QPSNSMSGNR-----------------------------PSH.NDPLQ.TGRV-----PLSVV--TAPPKR
Botrylloides-leachii_v3.S133.g02304.01.p_GH6-gene QNPE--------QYNQWVNQMRNGGSNTQSGSYGSQQQGSIPQSQPPNHQPSN---GNG-----------------------------RN..NDLAT.TGKV-----PISAI--TNPVRR
Molgula-oculata_S112948.g12660.01.p_GH6-gene NRPP--DPSDADAYNKYINQLNNNKN----------NDADLLNTQINSANNNQ---GN--ILLNNLLTAPAPRSQAIRTSPPRR.DVGQNN.D--...NDYVASNN-GLSATGYNNMMTS
Molgula-occidentalis_S285391.g07021.01.p_GH6-gene NRPQMNQINDIDAYNQQINSMNNNQA----------NNVNMQNTNRNNMNNNN---GNGGFMLTHYSTNRPVYSSTQRQS----.S.ESNN.D--...QGIVNRNGYGVSST---YPITS
Ciona-intestinalis_ENSCINT00000032307.1_GH6-gene ------------AQQQQVDRNNNN----------------FQQTSSPTATNSN---SN-------------------------RFRV---------------------------------
Oikopleura-dioica_AB543594.1_Od-CesA1-gene ------------------------------------------------YE-------------------------------YEEYGY--DYY-AQYDP----------------------
Oikopleura-dioica_AB543593.1Od-CesA2-gene -----------------------YGGGAYDAYGD--------------.GGD--AGGSD----------------------.GD.YA--..--GA..T----------------------
Oikopleura-longicauda_AB543515.1_Ol-CesA2-gene -----------------------YGGGDY--YGD-------------T.G-----AGDS----------------------.GG..D--...GGT...----------------------
Salpa-thompsoni_gbGFCC01072613.1.p1_CesA-gene -----------------------GGGGDYELPED-------------T..DG-----------------------------..AGDE..------VS-----------------------
Halocynthia-roretzi_AB543517.1_Hr-CesA-gene ------------------------------------------------------------------------------------------------------------------------
Botrylloides-leachii_v3.S157.g03251.01.p_CesA-gene -----------------------GGGGSNRGGGA-------------AAGGGGNVGGSD----------------------A--.SD--...-DA..DTS--------------------
Botryllus-schlosseri_008441g44194_CesA-gene ------------------------------------------------------------------------------------------------------------------------
Botryllus-schlosseri_008441g44193_CesA-gene ------------------------------------------------------------------------------------------------------------------------
Botryllus-schlosseri_008489g44332_CesA-gene ------------------------------------------------------------------------------------------------------------------------
Botryllus-schlosseri_008489g44331_CesA-gene ------------------------------------------------------------------------------------------------------------------------
Botryllus-schlosseri_008778g45080_CesA-gene -------------------SRGSDSGGG-------------------------------------------------------------...-NA.GDFD--------------------
Molgula-oculata_S71617.g04842.01.p_CesA-gene ------------AAG--------GGGGGAAGGGG-------------GDGGAAAAGGGD----------------------SSY.YDG-...-DG.ESDT--------------------
Molgula-oculata_v1-2.S69739.g04625.01.p_CesA-gene ---------------------WPGAGGS---------------------------GGVK----------------------PSD.DGGYY..--G..TI---------------------
Molgula-tectiformis_AB543516.1_Mt-CesA-gene ------------NAG------NTGGGGGNRGGGG-------------KVLGGGSVAADD----------------------SYA.TDGV...--G..GDS--------------------
Molgula-occidentalis_S469068.g15915.01.p_CesA-gene ------------------------------------------------------------------------------------------------------------------------
Molgula-occidentalis_S469068.g15913.01.p_CesA-gene -------------------------GGAN-------------------------------------------------------------------------------------------
Molgula-occidentalis_S469068.g15914.01.p_CesA-gene ------------A----------GGGGGGGGGGG-------------NAGGGKAAAGGE----------------------SYY.YDG-A..-GG..ADT--------------------
Ciona-savignyi_ENSCSAVT00000016381.1_CesA-gene ------------AADGGVGAAGGGAGGAAAGGGG-------------AAADNSAAAGTG----------------------GTD.YAGGE.T--A..STS--------------------
Ciona-intestinalis_BAD10864_CiCesA-gene ------------AAN--AGAAAGGAGGGAAAGNT-------------AAAGGGAAAGAA----------------------DTS.E.SY..T--AGGAT---------------------

Oikopleura-dioica_GSOIDT00010490001_GH6-gene ------SNKGLLDRETAEGIMKSISMKNNRMR..AKSSKMNY...YF.ARA...S......A.SV.K...T.GLT..GS..LITSV---------P...SGAPPMQQQ---GWGQPQPGW
Salpa-thompsoni_gbGFCC01117283.1.p1_GH6-gene -----VRSQFLLSRTEATGRMR--KKAASMK..IEKPSRNDAQ..NV...N...S....YNY.EFQP..ID.AVT.TGS.QMIA..--DKKSKP.E.D.KQKKSSGSD------------
Salpa-thompsoni_gbGFCC01119318.1.p1_GH6-gene -----------LTKSMASNKMS--ARTSTARL.MRKTSTN.AQ..NV.L....LA...M.GY.S..S..IG.IVQ......MLRYD--.MEQ---Y.Q.---------------------
Botryllus-schlosseri_botctg010729g9326_GH6-gene RP--TVKEVKLRTVQEALAIFR--NKITQRAR..QK.SL-SV..GNV.I..A..S....YEY....K...G..KL..KD.RLV.HG--DQR--SPV.G..KTNAVVNQ------------
Botrylloides-leachii_v3.S133.g02304.01.p_GH6-gene QP--T-RNVRLRAVQEAFEIFQ--RKSSQRFR..RK.AV-SV..GNV.I..A..S.....EY....K...G.RKQ..EK.RLAISP--.QR--PVS.G..RSNTPLSP------------
Molgula-oculata_S112948.g12660.01.p_GH6-gene QPGPITPASMLRSASVAGTMFG--AFMDKYQT.IRKNSV-QA..GNI.M.SA..A....YSY.S.RS..ID.NVM.T...KLL.GE--D.EM-PPQ.N..TPQTAAPPAVHGGG------
Molgula-occidentalis_S285391.g07021.01.p_GH6-gene GPGITSKPFSGRSKTAAGNLFG--TYMGNFAT.VRGNPV-.A..GNI.L..A..S...MYEY..F.K..IG.S.A.T.K.KML.VG--T.DM-PEE.D.EFSRPSPNP------------
Ciona-intestinalis_ENSCINT00000032307.1_GH6-gene -----------RTVAEASEMLR--SFRSRLRR.ATKPKF-A.N..NL.L.....S....YPY..FRS.....AQG...S.QMNQQR--DSNQ.PQQ.Q..S-------------------
Oikopleura-dioica_AB543594.1_Od-CesA1-gene ----------------------------AVLECLSNEI-EKGHDANSWVKTPGEGDGRLFASGTYHECLLEHTIECDDTCPAYVPKINGEFAREKMCTCDPDELLDYS------------
Oikopleura-dioica_AB543593.1Od-CesA2-gene ----------------------------G.IQ..TGDP-SG....NA.......A....YS...F.....Q.S...S.Q..Q.....N...E.PSG....X-------------------
Oikopleura-longicauda_AB543515.1_Ol-CesA2-gene ----------------------------V.....ANDP-SG....NA.I....GA...MYE...F.....G.AV..S...Q....SN...D.SDS.L..---------------------
Salpa-thompsoni_gbGFCC01072613.1.p1_CesA-gene ---------------------------DEDIV.ITTSE-.S.L..YA.......A.....P..EF.P..EG.SQD.S....Q....VA...Q.T.N.E.VDPKN----------------
Halocynthia-roretzi_AB543517.1_Hr-CesA-gene ------------------------------------------------------------------------------------------------------------------------
Botrylloides-leachii_v3.S157.g03251.01.p_CesA-gene -----------------------------.KA.V.PDQ-QE.L..FA.......S...M.P...F.P...G.LV..T.Q..Q....LS...Q.SD-------------------------
Botryllus-schlosseri_008441g44194_CesA-gene ------------------------------------------------------------------------------------------------------------------------
Botryllus-schlosseri_008441g44193_CesA-gene ------------------------------------------------------------------------------------------------------------------------
Botryllus-schlosseri_008489g44332_CesA-gene -------------------------------------------------------------------------------------.FN.KLX----------------------------
Botryllus-schlosseri_008489g44331_CesA-gene -------------------------------------------------------------------------------------------S.X--------------------------
Botryllus-schlosseri_008778g45080_CesA-gene ---------------------------VKLVS.V.PPD-QE.L..FV.......S...MYPA..F.Q...G.L...T...Q....LN.V.Q.SDS.R.IDAX------------------
Molgula-oculata_S71617.g04842.01.p_CesA-gene ----------------------------IAKA.V.K.G-.I.L.GYA.I.....S.....N.....P...N.Q...VE...Q....LN...Q.QES.S.KDE------------------
Molgula-oculata_v1-2.S69739.g04625.01.p_CesA-gene ----------------------------.AKA.V.K.G-.I.L.GYV.F.....S.....N.....P...N.Q...TA...QFM..LN...Q.QD-.S.KDE------------------
Molgula-tectiformis_AB543516.1_Mt-CesA-gene ---------------------------AVAKA.V.K.G-.T.L.GYA.I.....S.....N.....P...G.P...TE...Q.I..LN...Q.QES.G.KDEX-----------------
Molgula-occidentalis_S469068.g15915.01.p_CesA-gene ------------------------------------------------------------------------------------------------------------------------
Molgula-occidentalis_S469068.g15913.01.p_CesA-gene ------------------------------------------------LP.G.-------------------------------------------------------------------
Molgula-occidentalis_S469068.g15914.01.p_CesA-gene --------------------------DALAKA.V.N.G-.T.L.GYA.I.....A...I.P.....P...N.YV..TE...M....LN...Q.TES.H.KDE------------------
Ciona-savignyi_ENSCSAVT00000016381.1_CesA-gene ---------------------------D.AVI.FTA.QPGL.L..YA.......S...M.DA...P..TD.V.D.SE..SQ..T.VD.A.Q.SEA.Q.-SAPAA----------------
Ciona-intestinalis_BAD10864_CiCesA-gene ----------------------------.AVT.VTS.--AT.L..YA.I....S...M.DA...P..TD..T..SE..SQ..T.VD.A.Q.SEA.Q.GTVPEVX----------------
```

```
Oikopleura-dioica_GSOIDT00010490001_GH6-gene            GYQPN.MPQPQQQLNQWN----------P---QTWG-----------------------------------------------------
Salpa-thompsoni_gbGFCC01117283.1.p1_GH6-gene            -------.YSNAYDNYDN-----------------------------------------------------------------------
Salpa-thompsoni_gbGFCC01119318.1.p1_GH6-gene            -------------------------------------------------------------------------------VDF-------
Botryllus-schlosseri_botctg010729g9326_GH6-gene         -----NSRQPTQPPNNFNRGTVTQSSFFSPPSLQNSGVSSPSSQSQQSGGFNRDQSQILYSSNSADAEPQPAASGPPSLADLLRSGX--
Botrylloides-leachii_v3.S133.g02304.01.p_GH6-gene       -----FSQQPVQTSNSFNGGLFGQNA-------ENS-----QETDSDEXPFQGD-SQILYASNPVEPRSGPVQSGPPSLEDLLRQGR--
Molgula-oculata_S112948.g12660.01.p_GH6-gene            ----E.SAYPNQQQHYNAGGSPSLAQ---PNYNTNNGG----------GIFSNQESNS-YQQPYFN-------TAGSSLSDILQAAAGR
Molgula-occidentalis_S285391.g07021.01.p_GH6-gene       --------YANSMQNYNNRPNPYANT---P---PNSGGY--QNSNPRPSIFNNNNNQPLYPQSSYN-------TGVSSLSDLLQAAAGR
Ciona-intestinalis_ENSCINT00000032307.1_GH6-gene        -----------------------------------------------------------------------------------------
Oikopleura-dioica_AB543594.1_Od-CesA1-gene              -----YDEDVNYX----------------------------------------------------------------------------
Oikopleura-dioica_AB543593.1Od-CesA2-gene               -----------------------------------------------------------------------------------------
Oikopleura-longicauda_AB543515.1_Ol-CesA2-gene          -----------------------------------------------------------------------------------------
Salpa-thompsoni_gbGFCC01072613.1.p1_CesA-gene           -----------------------------------------------------------------------------------------
Halocynthia-roretzi_AB543517.1_Hr-CesA-gene             -----------------------------------------------------------------------------------------
Botrylloides-leachii_v3.S157.g03251.01.p_CesA-gene      -----------------------------------------------------------------------------------------
Botryllus-schlosseri_008441g44194_CesA-gene             -----------------------------------------------------------------------------------------
Botryllus-schlosseri_008441g44193_CesA-gene             -----------------------------------------------------------------------------------------
Botryllus-schlosseri_008489g44332_CesA-gene             -----------------------------------------------------------------------------------------
Botryllus-schlosseri_008489g44331_CesA-gene             -----------------------------------------------------------------------------------------
Botryllus-schlosseri_008778g45080_CesA-gene             -----------------------------------------------------------------------------------------
Molgula-oculata_S71617.g04842.01.p_CesA-gene            -----------------------------------------------------------------------------------------
Molgula-oculata_v1-2.S69739.g04625.01.p_CesA-gene       -----------------------------------------------------------------------------------------
Molgula-tectiformis_AB543516.1_Mt-CesA-gene             -----------------------------------------------------------------------------------------
Molgula-occidentalis_S469068.g15915.01.p_CesA-gene      -----------------------------------------------------------------------------------------
Molgula-occidentalis_S469068.g15913.01.p_CesA-gene      -----------------------------------------------------------------------------------------
Molgula-occidentalis_S469068.g15914.01.p_CesA-gene      -----------------------------------------------------------------------------------------
Ciona-savignyi_ENSCSAVT00000016381.1_CesA-gene          -----------------------------------------------------------------------------------------
Ciona-intestinalis_BAD10864_CiCesA-gene                 -----------------------------------------------------------------------------------------
```

CesA gene tree

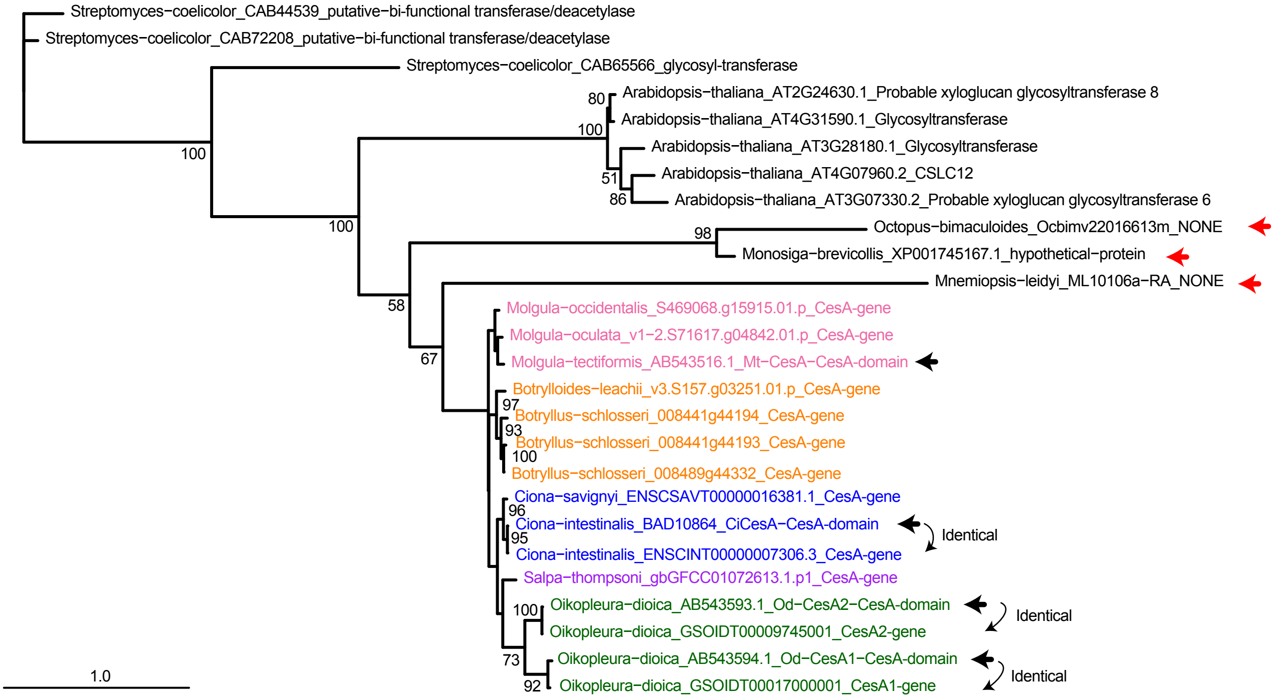

Fig. S2.

Phylogenetic position of the inconsistent *Monosiga brevicollis* sequence

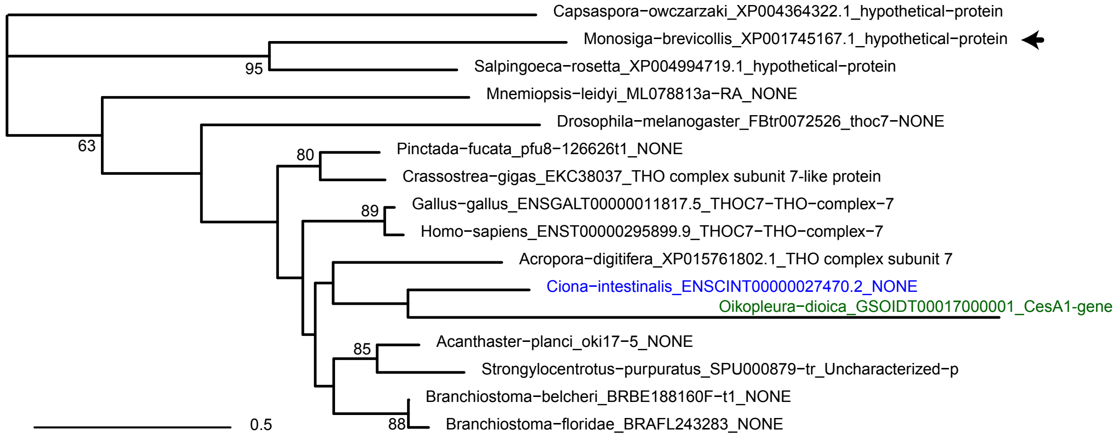

Fig. S3.
